# Diagnostic and prognostic significance of lncRNA SOX2-OT in patients with carotid atherosclerosis

**DOI:** 10.1186/s12872-022-02634-5

**Published:** 2022-05-10

**Authors:** Jianping Tao, Yu Hu

**Affiliations:** Department of Cardiology, Shanghai Sixth People’s Hospital Affiliated to Shanghai JiaoTong University, 600 Yishan Road, Shanghai, 200233 China

**Keywords:** Carotid atherosclerosis, SOX2-OT, Biomarker, Prognosis, Diagnosis

## Abstract

**Background:**

This paper aimed to analyze IncRNA SOX2-OT expression in patients with carotid atherosclerosis and to elucidate the predictive significance of SOX2-OT on carotid atherosclerosis.

**Methods:**

The levels of SOX2-OT from 185 participants were tested. The relationship between CIMT levels and SOX2-OT expression was examined by Pearson analysis. The clinical value of SOX2-OT was investigated by the ROC curve, K–M curve, and COX regression analysis. The comparison of SOX2-OT expression between patients with good prognosis and poor prognosis was also performed.

**Results:**

The expression of SOX2-OT was augmented in the patients with carotid atherosclerosis and was correlated with the level of CIMT. The high level of SOX2-OT might be a risk factor for carotid atherosclerosis. An enhancement of SOX2-OT expression was found in patients with poor prognosis. SOX2-OT might be an independent prognostic biomarker.

**Conclusions:**

SOX2-OT was upregulated in patients with carotid atherosclerosis and might be a predictive indicator in the progression of carotid atherosclerosis.

## Introduction

Atherosclerosis is a disorder formed by fibrofatty lesions in the artery wall [1]. Carotid atherosclerosis is the main manifestation of arteriosclerosis in the carotid artery. Atherosclerosis is one of major cardiovascular health threats. There is evidence that the mortality of ischemic heart disease (a clinical manifestation of acute coronary syndromes) is on the rise, especially in eastern Europe and Asia [2]. Cardiovascular disease led by atherosclerosis remains a major cause of vascular disease worldwide [3, 4]. Atherosclerosis is likely caused by chronic exposure to adverse life circumstances and environmental stressors, such as smoking, air contamination, sound pollution, less healthy dietary patterns, sleep deprivation, and psychosocial stress [5, 6]. In initiation and progression of atherosclerosis, stable plaques cannot produce any symptoms temporarily, but unstable plaques gradually progress and undergo repeated rupture and repair, leading to vascular stenosis and eventually acute cardiovascular events [1]. Although many cases have avoided premature death, they have to bear not only the burden of chronic cardiovascular diseases but also arthritis, depression, and other long-term obstacles to a healthy life [7]. Therefore, early identification is the key to effectively preventing cardiovascular events and death.

The regulatory effect of non-coding RNAs on atherosclerosis has been confirmed by many studies. Long non-coding RNA (lncRNA) MATERIAL, lncRNA MIAT, and lncRNA H19 can affect the processes of lipoprotein uptake, cholesterol reverse transport, cell inflammation, and angiogenesis [8–10]. MicroRNA (miR)-211-5p, miR-637, and miR-18a-5p can be used as novel biomarkers for the diagnosis of atherosclerosis [11–13]. Studies have also shown that lncRNA exists in peripheral circulating blood and can be used as a biomarker to reflect the severity of cardiovascular diseases and adverse prognosis. For instance, serum lncRNA-AWPPH is an independent risk factor for coronary artery disease and lncRNA THRIL is positively related to the accumulating rate of major adverse cardiovascular events in patients with coronary heart disease [14, 15]. An analysis manifests that the expression of lncRNA MALAT1 is elevated in humans with coronary atherosclerosis and has prognostic significance for in-stent restenosis [16]. LncRNA Sox2 overlapping transcript (SOX2-OT) is an lncRNA located in human chromosome 3q26.33 locus. In addition, lncRNA SOX2-OT levels are raised in hypoxic cell models of myocardial infarction and exacerbate cardiomyocytes injury, which provides that SOX2-OT may participate in the progress of vascular disease [17]. Thus, the significance of SOX2-OT in atherosclerosis arises our interest.

Our hypothesis was that SOX2-OT might play roles in atherosclerosis and possess clinical values for patients with atherosclerosis. Thus, we aimed to assess the clinical significance of SOX2-OT in atherosclerosis. For this purpose, the expression of SOX2-OT was evaluated in patients with atherosclerosis. The association between CIMT and SOX2-OT was provided by the Pearson correlation. The diagnostic role of SOX2-OT was tested by the ROC curve. The prognosis significance was unvealed by the K–M curve and Cox regression.

## Materials and methods

### Patients and sample processing

According to the inclusion criteria and exclusion criteria, 95 patients with asymptomatic carotid atherosclerosis from Shanghai Sixth People’s Hospital Affiliated to Shanghai JiaoTong University were selected for carotid ultrasound examination to evaluate the degree of carotid atherosclerosis. The ultrasonic diagnostic instrument model was ATL HDI 3000 ultrasound system (Advanced Technology Laboratories). All eligible patients meet the following inclusion criteria: carotid intima-media thickness (CIMT) of 0.9–1.2 mm [11]. The exclusion criteria were as follows: (1) coronary heart disease; (2) previous history of cerebrovascular disease; (3) severe dysfunction of organs such as heart, liver, and kidney; (5) pregnant women; (6) incorporation with the examination due to mental or other diseases; (7) thrombotic disease. The patients were first identified as asymptomatic carotid atherosclerosis and before this study, no patients took medicine. Besides, 90 healthy subjects from the same hospital were recruited as control group. This study obeys the ethical principles in the Declaration of Helsinki and has been authorized by the ethics committee of Shanghai Sixth People’s Hospital Affiliated to Shanghai Jiao Tong University. All papery informed consents were obtained from participants. The patient’s blood specimens were drawn from all patients in the morning after overnight fasting.

### Follow-up content

All participants were followed up for 5 years. The occurrence of adverse cardiovascular events, such as death, unstable angina pectoris, heart failure, and nonfatal myocardial infarction were recorded. The endpoint events were diagnostic according to the cardiovascular endpoint events guideline of ACC/AHA [18].

### RNA extraction and RT-qPCR

The total RNA was isolated from serum sample using the EZ-press serum/plasma RNA purification kit (EZBioscience). That is, the serum sample and the lysis solution were fully mixed and lysed, and the supernatant was collected. Then, the reagent and ethanol were mixed and transfer to an RNA binding column. The eluent reagent was used to remove impurities efficiently and purify total RNA by subsequent washing.

The gDNA remover (EZBioscience) was utilized to remove the genomic DNA residues in the RNA extraction process, and the treated RNA samples were directly used for subsequent experiments. The reverse transcription system (GenStar, Beijing, China) was used to synthesize first-strand cDNA from the total RNA. Then, the realstar green fast mixture (GenStar, Beijing, China) was used for assessed relative expression of SOX2-OT. That is, 50 ng cDNA was mixed with 1 µl primers (10 µM), 25 µl 2×RealStar Fast SYBR qPCR Mix, 1 µl ROX Reference Dye, and Sterile Water. After that, this mixture was put on the ABI 7500 system. The expression of serum GAPDH was detected as an internal reference. The expression was processed using the 2^−ΔΔCt^ formula.

### Statistical analysis

According to 5% of false positive error rate (two-sided, alpha = 0.05), power = 90%, beta = 0.1, and dropout rate = 20%, PASS 11.0 was utilized to estimate the sample size, and at least 70 subjects in each group should be the sample size limit. All the data were analyzed by SPSS 26.0 statistical software. T-test was used to compare the measurement data between groups, χ^2^ test was used for counting data. The clinical values of SOX2-OT were predicted by the Pearson correlation, ROC curve, K–M curve following log-rank test, and COX regression test. COX regression analysis was used to analyze the influencing factors of atherosclerosis. Among them, *P* < 0.05 was thought that the difference was statistically significant.

## Results

### Comparison of basic characteristic data

The age of 95 patients with carotid atherosclerosis was 60.01 ± 7.83 years old (Table 1). There were 56 males and 39 females in the carotid atherosclerosis group (Table 1). This study showed that the proportion of hypertension patients in the atherosclerosis group was higher, and the items of LDL-C, CRP, and CIMT in the atherosclerosis group were also higher than those in control group (Table 1, *P* < 0.05). There were no significant differences on age, sex, drinking, smoking, diabetes, and HDL-C between the two groups (Table 1, *P* > 0.05).

**Table 1:** Clinical baseline data of the study subjects

Items	Control (n = 90)	Carotid atherosclerosis (n = 95)	*P* values
Age, years	59.71 ± 7.49	60.01 ± 7.83	0.791
Sex, male/female	45/45	56/39	0.222
Drinking, n			0.143
Yes	25	36	
No	65	59	
Smoking, n			0.245
Yes	35	45	
No	55	50	
Hypertension, n			0.049
Yes	41	57	
No	49	38	
Diabetes, n			0.183
Yes	33	44	
No	57	51	
HDL-C (mmol/ml)	1.20 ± 0.18	1.15 ± 0.18	0.098
LDL-C (mmol/ml)	2.75 ± 0.62	3.44 ± 0.87	< 0.001
CRP (mg/l)	5.08 ± 3.42	14.55 ± 3.30	< 0.001
CIMT (mm)	0.49 ± 0.18	1.09 ± 0.08	< 0.001

HDL-C, high-density lipoprotein; LDL-C, low density lipoprotein; CRP, C-reactive protein; CIMT, carotid intima-media thickness. Data are expressed as n or mean ± standard deviation

### Expression of SOX2-OT in atherosclerosis

**Fig. 1:**
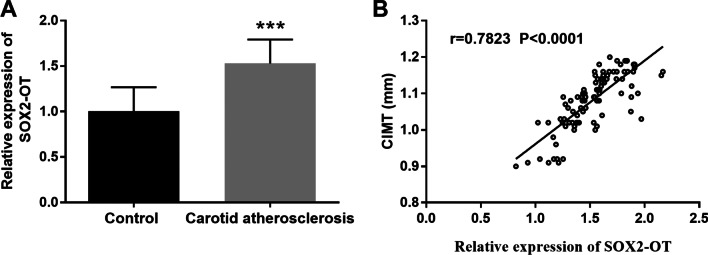
The expression of SOX2-OT and its relationship to CIMT. **A** The expression of SOX2-OT was increased in patients with asymptomatic carotid atherosclerosis. **B** The level of CIMT was related to SOX2-OT expression. ***P < 0.001

All serum specimens of participants were applied in the RT-qPCR to analyze the expression of SOX2-OT. The serum SOX2-OT level in the atherosclerosis group was significantly higher than that in the control group (Fig. 1A, *P* < 0.001).

### Connection of SOX2-OT and CIMT

Based on the abnormality of SOX2-OT expression in patients with atherosclerosis, Pearson association analysis was performed to elucidate the interconnection between SOX2-OT expression and CIMT levels. As shown in Fig. 1B, the relative levels of SOX2-OT were pertinent to the imaging marker CIMT, discerning that SOX2-OT might be implicated in atherosclerosis (r = 0.782, *P* < 0.001).

### Diagnostic performance of SOX2-OT

**Fig. 2:**
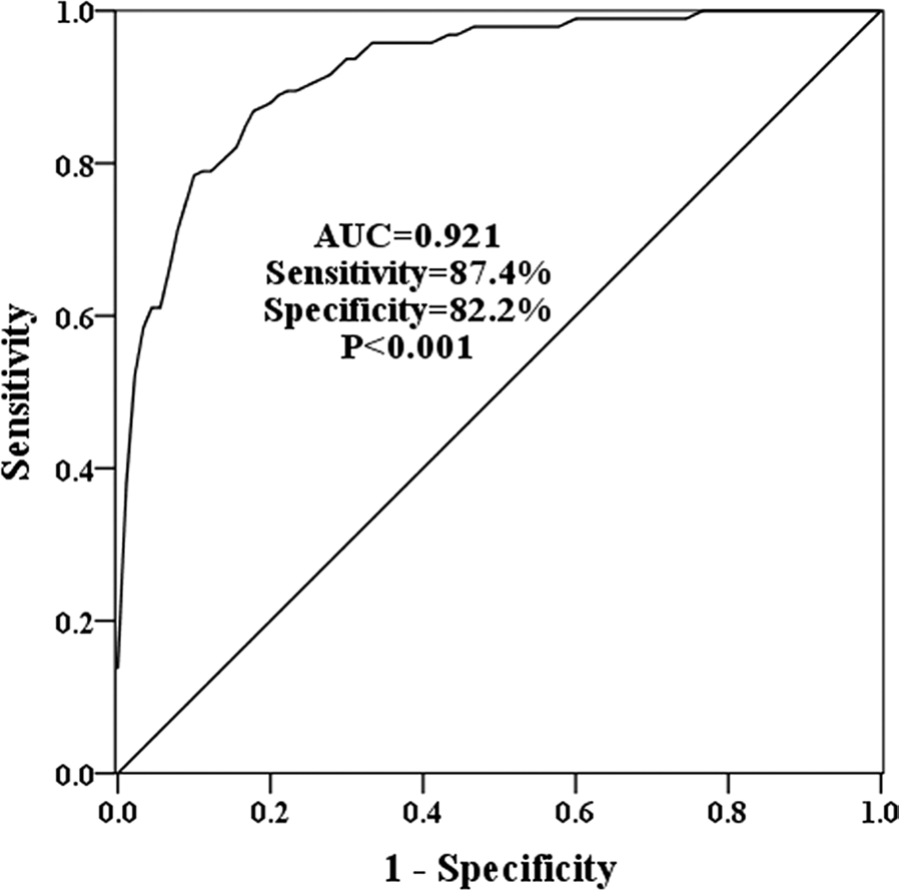
The predictive role of SOX2-OT. The AUC of SOX2-OT was 0.921 with the sensitivity of 87.4% and the specificity of 82.2%

The possible utility of serum SOX2-OT as a biomarker for atherosclerosis patients was evaluated. As exhibited in Fig. 2, the AUC of SOX2-OT was 0.921, and the sensitivity of 87.4%, specificity of 82.2% at the cutoff value of 1.25. This discovery indicated that SOX2-OT might serve as a clinical marker.

### Prognostic role of SOX2-OT

**Fig. 3:**
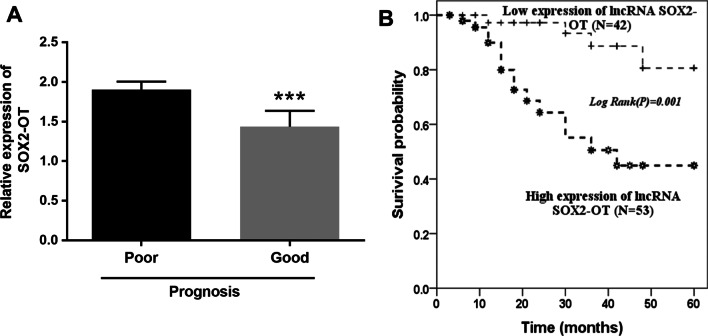
The roles of SOX2-OT on prognosis. **A** The relative expression of SOX2-OT in patients with good outcomes was decreased compared with that in patients with poor outcomes. The relative expression level of SOX2-OT was normalized with GAPDH. **B** The patients with high expression of SOX2-OT had high incidence of adverse cardiovascular events. ***P < 0.001

All patients were divided into poor outcomes and good outcomes according to the situation of a 5-year follow-up. There were 19 patients with endpoint events, and 76 patients without. The expression of SOX2-OT was declined in the good prognosis patients compared with the poor prognosis patients (Fig. 3A, *P* < 0.001).

The prognostic importance of serum SOX2-OT in patients with atherosclerosis was tested by K–M analysis. A total of 42 patients was divided into low expression group and 53 patients were recruited in the high expression group in accordance with the median expression of SOX2-OT. The finding showed that the occurrence of endpoint events in the low expression of SOX2-OT was lower than that in the high expression of the SOX2-OT group (Fig. 3B, *P* = 0.001).

### Possibility of SOX2-OT as an independent biomarker

In COX regression analysis, the incidence of cardiovascular events in five years was taken as dependent variables, and age, sex, drinking, smoking, hypertension, diabetes, HDL-C, LDL-C, CRP, CIMT, and serum SOX2-OT were taken as independent variables. The results showed that CRP (95% CI = 1.129–15.157, HR = 4.137, *P* = 0.032), CIMT (95% CI =1.104–11.932, HR = 3.629, *P* = 0.034), and SOX2-OT (95% CI = 1.200–13.984, HR = 4.096, *P* = 0.024) were independent risk factors for patients with atherosclerosis (Table 2).

**Table 2:** Multivariate Cox analysis of independent factors affecting cardiovascular endpoint events

Measurements	95% CI	HR	*P* values
Age	0.529–4.705	1.578	0.413
Sex	0.373–3.897	1.206	0.754
Drinking	0.385–3.955	1.233	0.724
Smoking	0.392–3.352	1.146	0.804
Hypertension	0.487–3.903	1.378	0.546
Diabetes	0.472–7.293	1.854	0.377
HDL-C	0.144–1.427	0.453	0.176
LDL-C	0.694–5.444	1.943	0.206
CRP	1.129–15.157	4.137	0.032
CIMT	1.104–11.932	3.629	0.034
LncRNA SOX2-OT	1.200-13.984	4.096	0.024

HDL-C, high-density lipoprotein; LDL-C, low density lipoprotein; CRP, C-reactive protein; CIMT, carotid intima-media thickness

## Discussion

Asymptomatic carotid atherosclerosis, which has no history of cardio-cerebral vascular events, is an early stage of atherosclerotic disease [19]. Carotid atherosclerosis can reflect the degree of systemic atherosclerosis and is one of the main causes and independent risk factors of stroke, dementia, and cardiovascular events [20, 21]. In recent years, the improvement of residents’ living standards has led to the refinement of diet structure, the formation of unhealthy living habits, and the increase of work pressure, which has led to the increased risk of cardiovascular events, among which carotid atherosclerosis is the most prominent. The nature and degree of stenosis of internal carotid atherosclerotic plaque will have an impact on cardiovascular events. Thus, early identification and intervention are of great significance.

LncRNAs are widely distributed in animals, plants, viruses, and other organisms, and they can play roles in gene expression regulation, cell proliferation, apoptosis, and metabolism. Many studies have confirmed that IncRNAs can affect the occurrence and development of atherosclerosis at the epigenetic level. In this paper, SOX2-OT was highly expressed in the patients with carotid atherosclerosis, suggesting that carotid atherosclerosis might lead to the alternation of SOX2-OT expression. In addition, the elevated SOX2-OT was correlated with the increased CIMT levels, which suggested that the progression of carotid atherosclerosis might contribute to the abnormality of SOX2-OT expression. Accumulated evidence reports the crucial roles of SOX2-OT in the regulation of tumors and various diseases. It is reported that the expression of SOX2-OT is enhanced in lung cancer, gastric cancer, and esophageal cancer [22–24], which indicates that SOX2-OT is a oncogene in cancers. The expression of SOX2-OT is raised during central nervous system development and ischemic heart failure [25, 26], providing the relationship between SOX2-OT and cardiovascular events. As summarized previously, SOX2-OT might participate in the development of atherosclerosis.

In the present report, the clinical significance of SOX2-OT was further revealed. The finding of ROC curve implied that SOX2-OT might be a biomarker to differentiate the patients with carotid atherosclerosis from healthy cohorts with desirable specificity and sensitivity. In addition, the expression of SOX2-OT in the patients with good outcomes was reduced, which revealed that the expression of SOX2-OT might change with the development of carotid atherosclerosis. Besides, the K–M analysis discovered that patients with high SOX2-OT levels had a high possibility of suffering cardiovascular events, indicating that overexpression of SOX2-OT is a risk of poor outcomes. In addition, the result of Cox regression analysis indicated that SOX2-OT might be an independent prognostic biomarker in predicting the outcome of patients with carotid atherosclerosis. As reported by previous publications, several lncRNAs have the probability of being markers in chronic vascular diseases. In coronary atherosclerosis, Qual et al. discern that PVT1 is an independent factor influencing disease development [27]. The ROC curve of lncRNA HIF1A-AS1 propounds that this lncRNA may serve as an indicator for patients with atherosclerosis [28]. All these data suggested that lncRNA might act as a biomarker in the progression of vascular dysfunction. However, the limitations of small sample size and short duration of follow-up hampered the appliance of SOX2-OT. Limitations also include the lack of positive control lncRNA, lack of data comparing the SOX2-OT expression before and after treatment in patients, without usage of pulse wave velocity. More investigates would be performed on larger population and heterogenous population.

## Conclusions

In total, an enhancement of SOX2-OT expression in patients with asymptomatic carotid atherosclerosis was evaluated. The upregulation of SOX2-OT was associated with the ascended CIMT levels. Moreover, SOX2-OT might be a predictive indicator of the identification and progression of carotid atherosclerosis. This study discovered a novel biomarker in carotid atherosclerosis and this finding might be beneficial to the screen and predict patients with asymptomatic carotid atherosclerosis.

## Data Availability

The datasets generated during the current study are not publicly available due this study is only a part of the research direction of the authors’ research group, and we will continue to carry out the following research, but are available from the corresponding author on reasonable request.
